# Correlation of lower 2 h C-peptide and elevated evening cortisol with high levels of depression in type 2 diabetes mellitus

**DOI:** 10.1186/s12888-020-02901-9

**Published:** 2020-10-06

**Authors:** Yu Ming Sang, Li Jun Wang, Hong Xian Mao, Xue Yong Lou, Yi Jun Zhu, Yue Hua Zhu

**Affiliations:** 1 Department of Endocrinology, Jinhua Central Hospital, 351 Mingyue Street, Jinhua City, 321000 Zhejiang Province China; 2 Department of Psychology, Zhejiang Normal University, 688 Yingbin Road, Jinhua, 321004 Zhejiang Province China; 3 The Central Laboratory, Jinhua Central Hospital, 351 Mingyue Street, Jinhua City, 321000 Zhejiang Province China; 4 Department of Psychiatry, Jinhua Central Hospital, 351 Mingyue Street, Jinhua City, 321000 Zhejiang Province China

**Keywords:** C-peptide, Cortisol, Depression, Ghrelin, Leptin, Middle-aged

## Abstract

**Background:**

A number of studies have explored the association between depression and ghrelin, leptin, and cortisol; further, postprandial C-peptide levels have a therapeutic effect on type 2 diabetes mellitus (T2DM). However, the relationship between C-peptide and depression in patients with diabetes, remains unclear. The aim of this study was to explore the association between depression and ghrelin, leptin, cortisol, and C-peptide in patients with diabetes.

**Methods:**

We enrolled 50 adults without T2DM, 77 non-depressed adults with T2DM (free of Axis-I psychiatric disorders as assessed using the Mental Illness Needs Index (MINI), Patient Health Questionnaire (PHQ-9 score ≤ 4)) and 59 patients with T2DM and depression (PHQ-9 ≥ 7 and positive by the Structured Clinical Interview for DSM-5). The age range of the participants was 45–59 years of age. We compared the above three groups and explored the association between ghrelin, leptin, cortisol, C-peptide, and depression in patients with diabetes. A post-hoc power-analysis was finished.

**Results:**

Compared with the non-depression T2DM group, the depression T2DM group had significantly higher blood glucose fluctuations. Further, compared with the non-depression T2DM and non-diabetic groups, the depression T2DM group had significantly lower levels of post-meal 2-h C-peptide and elevated evening cortisol (*p* < 0.01). Regression analysis revealed a significant negative correlation between depression severity and 2-h postprandial C-peptide in patients with diabetes (*p* < 0.01) and a significant positive correlation with midnight cortisol levels (*p* < 0.01). A post hoc power analysis showed that we had an adequate sample size and met the minimum requirement to attain 80% power. A post hoc power calculation also demonstrated that this study basically achieved power of 80% at 5% alpha level.

**Conclusions:**

Our findings indicate a correlation of low fasting levels of 2-h C-peptide as well as higher midnight cortisol levels with higher depression severity in middle-aged patients with T2DM.

## Background

Depression and diabetes are individually very disabling disorders; they are currently ranked the 11th and 14th leading causes of disability-adjusted life years in the global burden of disease study, respectively [1]. Recent studies show that the most commonly occurring comorbidities are depression and diabetes [2]. As of 2010, over 100 million adults in China were diagnosed with diabetes mellitus; approximately 10% of adults over 18 years of age had diabetes [3]. Many studies have demonstrated an increased prevalence of depressive symptoms and major depressive disorder in patients with diabetes [4]. Previous studies have also explored factors that affect depression in patients with diabetes mellitus and showed that poor control of diabetes is one of the important factors precipitating depression [5–10]. However, the physiological reasons for controlling blood sugar are not well understood.

C-peptide (CP) is used to evaluate islet function in patients with type 2 diabetes mellitus (T2DM) and to measure the extent of endogenous insulin deficiency [11]. The increase in postprandial 2 h C-peptide (2hCP) is proportional to the degree of improvement seen in patients after intensive treatment [12]. Previous studies demonstrate that patients with insulin-treated T2DM, but low postprandial C-peptide levels, have markedly increased glucose variability and incidence of hypoglycemia and hyperglycemia compared with those who retained C-peptide [13]. Measured by continuous glucose monitoring or self-report, hypoglycemia or severe hyperglycemias are able to induce negative emotional states in patients with diabetes [14]. Based on prior studies, the hypotheses of this study are that postprandial C-peptide levels are lower, blood glucose fluctuations are greater, and levels of depression-related symptoms are higher in diabetic patients.

Ghrelin is a peptide secreted in the stomach that promotes eating, whereas leptin is secreted by fat cells and acts as an appetite inhibitor. These two peptides regulate food intake, control body weight, and regulate energy balance by acting on the hypothalamus [15, 16]. Ghrelin levels in the blood dynamically change and are increased during starvation and decreased after meals [17]. In addition to regulating feeding, ghrelin and leptin play a role in mood regulation since ghrelin and leptin receptors are found in many brain regions, including the hippocampus and amygdala [18–22]. Moreover, Poretti et al. reported that ghrelin regulates depression-related behavior by affecting gene expression [23]. There has been a recent increase in studies on the relationship between ghrelin, leptin, and depression; however, they have not reported consistent findings [24–32]. Changes in cortisol levels occur depending on physical and psychological stress levels, as well as circadian rhythms, which reflects the activity pattern in the hypothalamic-pituitary-axis [33]. This was previously thought to positively correlate with depression levels [34]. Thus, the hypothesis of this study is that dysregulation (either high or low levels) of ghrelin and leptin are associated with the level of depression symptoms in individuals with diabetics, while cortisol is positively correlated with depression symptoms.

To further explore changes in the above variables in patients with comorbid depression and diabetes, the study also included assessments in non-diabetic individuals. After matching for sex and age, differences between depressive patients with diabetes, non-depressive patients with diabetes, and non-diabetic individuals were measured and analyzed.

## Methods

### Participants

**Fig. 1:**
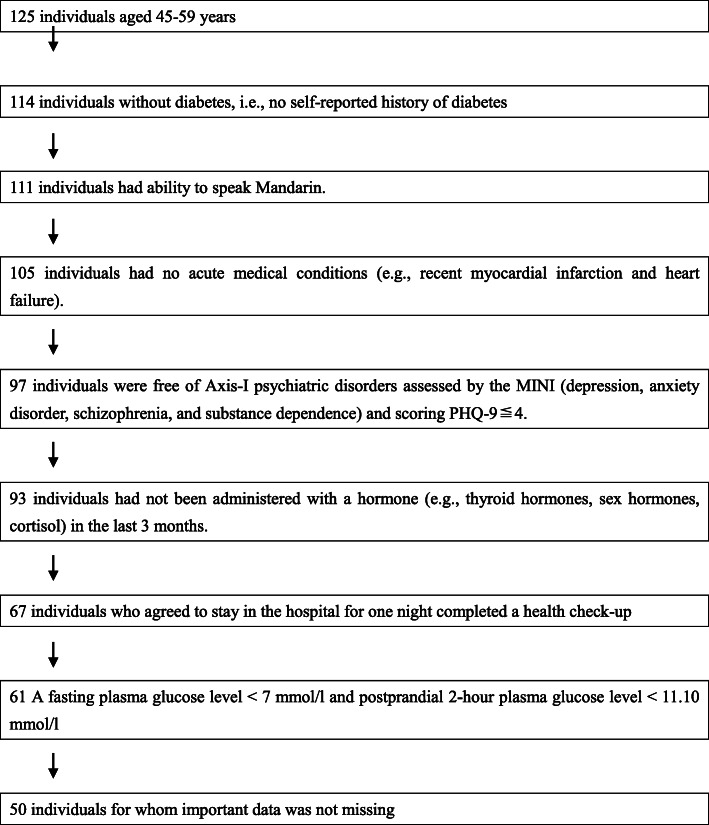
Flow diagram showing the selection of the non-diabetic study individuals

A cohort of 186 adults (45–59 years old) including 136 with diabetes (88 men and 48 women) and 50 without diabetes (31 men and 19 women) participated in the study. Individuals without diabetes were community residents recruited through advertisements. The process for selecting individuals without diabetes for this study is shown in Fig. 1.

All study participants underwent structured psychiatric interviews (MINI) [35] performed by an experienced psychiatrist. The MINI was designed to meet the need of a short but accurate and structured psychiatric interview for multicenter clinical trials and epidemiological studies. All non-diabetic and non-depressed individuals were free of Axis-I psychiatric disorders (depression, anxiety disorder, schizophrenia, and substance dependence), as assessed by the MINI. Depression was further assessed using the Patient Health Questionnaire-9 (PHQ-9) [36, 37] and the Centre for Epidemiologic Studies Depression scale (CES-D) [38]. Only individuals scoring four or less on the PHQ-9 were included in either the non-diabetic group or the non-depressed group.

Individuals with diabetes were selected from inpatients with insulin-treated T2DM, and met the following criteria: (a) had a fasting blood glucose level of > 7 mmol/l, on two separate occasions, (b) had a 2-h blood glucose level of > 11.10 mmol/l, during an oral glucose tolerance test (75 g) on two separate occasions, and (c) a prior diagnosis of T2DM. First, diabetic patients were excluded who had large blood glucose level fluctuations and recurrent hypoglycemia or hyperglycemia. Other exclusionary criteria were as follows: those with a history of hormone administration (e.g., thyroid hormones, sex hormones, and cortisol) in the last 3 months; low visual acuity or serious hearing problems; inability to speak Mandarin; acute medical conditions (e.g., recent myocardial infarction and heart failure); and a history of significant neurological or psychiatric disorders (e.g., AD, stroke, schizophrenia). Hospitalized individuals with diabetes who met the above criteria voluntarily participated in this study at the Jinhua Central Hospital between January 2016 and January 2018. This study was approved by the ethics committee of the Central Hospital of Jinhua and the academic committee of Zhejiang Normal University. Written informed consent was obtained from each individual before the study.

### Measures

#### Depression test

All participants, including non-diabetic individuals and patients with T2DM, took depression evaluation tests. A total of 223 patients with T2DM were tested, and 29 patients with missing critical data were excluded. The level of depression was measured using the PHQ-9 [39, 40] and the CES-D [38]. PHQ-9 depression severity is calculated by assigning scores of 0, 1, 2, and 3, to the response categories of “not at all,” “several days,” “more than half the days,” and “nearly every day”, respectively. The total score for the nine items ranges from 0 to 27. Zhang et al. reported the validity of PHQ-9 for depression screening in Chinese patients with T2DM, with a lower cutoff point (≥7) for depressive symptoms [39, 40]. The CES-D scale is a 20-item self-reported instrument developed by Radloff in 1977 [38]. It measures the frequency of common depression-related symptoms over the most recent week. Each item is scored from 0 (rarely or none of the time, less than 1 day) to 3 (all the time, 5–7 days). The four positively stated items are reverse-coded for calculating the total score, which ranges from 0 to 60. Therefore, only those scoring higher (≥7) on the PHQ-9 were included as positive individuals; positive screens were followed-up by the Structured Clinical Interview for DSM-5 [41]. According to the above standards, 69 of the patients were included in the depression with diabetes group. To prevent drug treatment as a confound impacting hormone levels, ten patients who were actively on antidepressants were excluded. Ultimately, 59 of the patients were included in the depression with diabetes group. Only individuals scoring low on the PHQ-9 (≦4) were included in the non-depression group. According to the above standards, 77 patients were included in the non-depression diabetes group, and 50 adults were included as non-diabetic participants. This study used the Chinese version of the PHQ-9 as a screening tool for depression and the CES-D scale to check the criterion-related validity.

#### Evaluation of physiological indices

After patients with diabetes had completed the formalities of hospitalization on the next day and following a 10-h overnight fast (i.e., the participants fasted from 10:00 pm), blood samples were obtained at 8:00 am, 10:00 am, 4:00 pm, and midnight; further, 24-h urine samples were obtained. Participants ate a uniform breakfast, specially designed for diabetics by the hospital nutrition department, immediately after blood samples were taken at 8:00 am. Plasma samples were stored at − 80 °C for subsequent analysis. Leptin and total ghrelin plasma levels were analyzed using radioimmunoassay kits (Anhui Anke Biotechnology Ltd., by Share Ltd.; Phoenix Pharmaceuticals, Inc., USA California). Cortisol levels were determined through chemiluminescence using commercially available LN CLIA Microparticles kits (Autobio Diagnostics Co. Ltd., Zhengzhou, China). Each participant’s height and weight were measured and used to calculate the body mass index (BMI). The kits used for ghrelin and leptin analyses were similar to those used in our previous studies [42].

Serum C-peptide levels were analyzed using an Abbott Axsym chemiluminescence immune analyzer (Abbott Laboratories, Abbot Park, IL, USA). Hemoglobin A1c (HbA1c) levels were measured by high-performance liquid chromatography. Diabetic patients wore a dynamic glucose monitoring system for 72 h (DGMS, purchased from San MediTech medical technology co., Ltd. China, Huzhou). Recorded and analyzed were the average daytime blood glucose (MBG), average daytime blood glucose fluctuations (MAGE), standard deviation of blood glucose (SDBG), and maximum blood glucose fluctuation (LAGE).

### Analyses

Normal data are expressed as the mean ± standard deviation, and non-normal data are expressed as median and interquartile range. For subsequent analysis of variance (ANOVA) and regression analysis, the variables of continuous data were tested by normal distribution using the Kolmogorov-Smirnov Test. The results show that education level, duration of diabetes, and fasting leptin levels do not satisfy a normal distribution; they were therefore log-transformed. The Kolmogorov-Smirnov Test performed again after the conversion indicates that the three transformed variables conform to a normal distribution. Continuous data among the three groups were compared using a one-dimensional analysis of variance (ANOVA). Subsequently, Bonferroni post hoc tests were used to compare the average difference. Regarding two columns of continuous data, the independent or paired-sample t test was used depending on the method for data fitting. The Chi-square test was used for counting data. Cortisol data obtained at three time points were analyzed using a mixed-design variance analysis with the three time points as the within-participant variable, the 3 groups (groups: non-diabetic individuals, depression individuals with diabetes, and non-depression individuals with diabetes) as the between-participant variable, and BMI as the covariate.

A post-hoc power analysis using G*Power 3.1.9 was used to calculate the overall statistical power of the present study [43, 44]. A post hoc power calculation was also conducted to estimate the minimum sample size required to achieve a power of 80% at 5% alpha level.

#### Regression models

In the multiple stepwise regression model, the depressive scores were used as the dependent variables. The following independent variables were used: age; gender; BMI; years of education; duration of diabetes; history of hypertension; FBG; HbA1c; fasting ghrelin and leptin levels; plasma cortisol levels at 8:00 am, 4:00 pm, and 12:00 pm; 24-h urinary cortisol levels; FCP; and 2hCP. Based on the importance of each independent variable, each step selects a variable for entry into the regression equation. This is followed by alternatingly adding and removing independent variables until no new significant variables can be introduced and no non-significant independent variables can be removed. Finally, the independent variables that remain in the regression equation are considered to be significantly related to the dependent variables.

## Results

### Comparison between non-diabetic, diabetic depression and diabetic non-depression groups

A minimum sample size of 84 and 102 participants was required to attain 80% power at 5% alpha level for correlation tests (two tailed) and Independent sample t tests, respectively; therefore, our sample size of 136 patients with T2DM was adequate. As a minimum sample size of 159 and 108 participants was required to attain 80% power at 5% alpha level for one dimensional analysis of variance and chi-square tests, respectively, our sample size of 186 persons overall was also adequate. Both correlation tests achieved a power of 1 (the correlation values were 0.382 and 0.441, 136 patients with T2DM as samples). The post-hoc power analysis was conducted for 24 tests from Table 1. The results demonstrated that the powers of 11 tests were between 0.80 and 0.93, the power of eight tests were between 0.70 and 0.79, the powers of five tests were between 0.35 and 0.68, and average power was 0.78. The overall statistical power of this study (based on the post-hoc power analysis) was acceptable (78%). The post hoc power analysis showed that our tests meet the requirements of power = 0.8.

**Table 1:** Comparison between the two diabetic (depression and non-depression) and non-diabetic groups

	Non-diabetic subjects	Diabetic subjects		
		Depressive	Non-depressive	
	PHQ-9≦4	PHQ-9≧7	PHQ-9≦4	
	*n* = 50	*n* = 59	*n* = 77	p
Age(years)	52.23 ± 8.50	53.12 ± 6.80	51.45 ± 7.66	0.215
Years of education, median (IQR)	8(6, 16)	8(8, 10)	8(6, 11.5)	0.250
Female/male	19/31	18/41	30/47	0.562
BMI(kg/m^2^)	24.16 ± 4.74	25.69 ± 4.38	24.25 ± 4.99	0.113
Duration of diabetes(years)		2(0.9, 7.8)	2(0.8, 6.9)	0.392
History of hypertension (yes/no)	8/42	20/39	22/55	0.100
**FBG(mmol/l)**	**5.25 ± 0.28**^**a**^	**10.33 ± 3.49**	**9.56 ± 2.52**^**b**^	**0.000**
**PBG(mmol/l)**	**6.41** ± **1.85**^**a**^	**12.48 ± 4.98**	**11.91 ± 4.08**^**b**^	**0.000**
**HbA1c(%)**	**5.35** ± **0.48**^**a**^	**9.86 ± 2.89**	**9.52 ± 2.23**^**b**^	**0.000**
**FCP(nmol/l)**	**0.47** ± **0.18**^**a**^	**0.61 ± 0.49**	**0.53 ± 0.19**	**0.018**
**2hCP(nmol/l)**	**1.51** ± **0.66**^**a**^	**1.03 ± 0.47**^**c**^	**1.46 ± 0.64**	**0.001**
**MBG(mmol/l)**		**7.89 ± 1.59**	**7.64 ± 1.35**	**0.708**
**MAGE(mmol/l)**		**5.95 ± 1.87**^**c**^	**4.41 ± 1.63**	**0.000**
**SDBG(mmol/l)**		**2.76 ± 1.79**^**c**^	**1.90 ± 1.43**	**0.000**
**LAGE(mmol/l)**		**10.66 ± 3.41**^**c**^	**8.12 ± 3.49**	**0.000**
24-h urine cortisol (μg/dl)	226.98 ± 299.45	320.41 ± 311.74	230.49 ± 337.57	0.079
8:00 am Plasma cortisol (μg/dl)	15.59 ± 4.64	16.90 ± 8.85	15.93 ± 6.66	0.570
4:00 pm Plasma cortisol (μg/dl)	8.96 ± 4.45	11.14 ± 9.73	8.68 ± 4.23	0.101
**12:00 pm Plasma cortisol (μg/dl)**	**3.41 ± 3.39**^**a**^	**7.88 ± 5.67**^**c**^	**5.61 ± 4.57**^**b**^	**0.000**
**Fasting ghrelin(ng/ml)**	**11.84 ± 1.57**^**a**^	**9.67 ± 1.93**^**c**^	**11.47 ± 1.62**	**0.001**
Fasting leptin(ng/ml)	10.23(9.71, 14.92)	11.79(7.46, 15.20)	10.70(6.08, 14.03)	0.467
**Ghrelin changes(ng/ml)**	**−0.94 ± 1.09**^**a**^	**−0.40 ± 0.97**	**−0.61 ± 0.80**	**0.022**
PHQ-9	**1.92 ± 0.95**^**a**^	**8.94 ± 2.35**^**c**^	**2.05 ± 1.07**	**0.000**
CES-D	**4.31 ± 3.52**^**a**^	**12.33 ± 6.23**^**c**^	**6.01 ± 3.48**	**0.000**

*PHQ-9* Patient Health Questionnaire-9, *BMI* Body mass index, *FBG* Plasma glucose before the meal, *HbA1c* Glycated hemoglobin, *PBG* Postprandial blood glucose, *FCP* Fasting C-peptide, *2hCP* 2 h postprandial C-peptide MBG, the day time mean values blood glucose, *MAGE* Mean amplitude of *glucose* excursions, *SDBG* Standard deviation blood glucose, *LAGE* Large amplitude of glycemic excursions, *ghrelin changes* the changes before and after meals, *CES-D* Center for Epidemiologic Studies Depression Scale, *IQR* Interquartile range

*p*-value: One dimensional analysis of variance (ANOVA) significance level when comparing three groups above; chi-square (*x*^2^) significance level for categorical variables. Independent sample t test significant level for two independent samples

^a^There was a significant difference between the non-diabetic and the depressive subjects with diabetes

^b^There was a significant difference between the non-diabetic and non-depressive subjects with diabetes

^c^There was a significant difference between the non-depressive and depressive subjects with diabetes

**Fig. 2:**
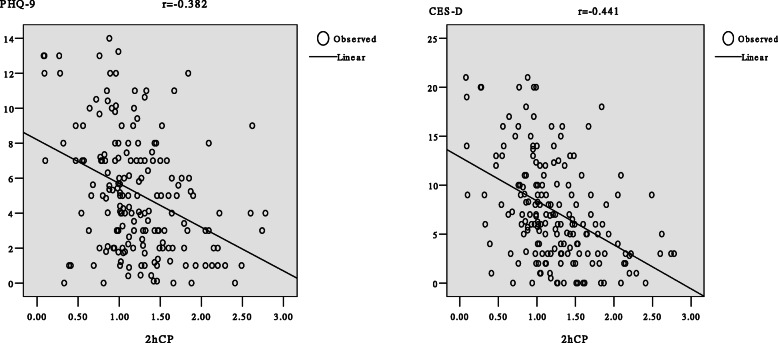
Scatter diagram and correlation coefficient of association of PHQ-9 and CES-D scores with 2hCP

The PHQ-9 and CES-D showed high internal consistency with a Cronbach’s alpha of 0.83 and 0.89, respectively. Because of the high internal consistency of both surveys, all items could be combined to calculate the total scores, thus representing the overall level of depression. The Pearson’s correlation coefficients of PHQ-9 and CES-D scores were 0.816; that between 2hCP and PHQ-9 scores was − 0.382, while that between 2hCP and CES-D scores was − 0.441. The scatter plots showing the correlations between PHQ-9 and CES-D scores and 2hCP in patients with T2DM are shown in Fig. 2. PHQ-9 and CES-D scores in depressive individuals with T2DM were significantly higher than in those in either the non-depression T2DM group or the non-diabetic group (*p* < 0.001, Table 1).

As seen in Table 1, ghrelin baseline levels were significantly lower in the depression with T2DM group (9.67 ± 1.93 ng/ml) compared with either the non-depression T2DM (11.47 ± 1.62 ng/ml) or non-diabetic (11.84 ± 1.57 ng/ml) groups. FCP was significantly higher in the depression and T2DM group (0.61 ± 0.49 nmol/l) compared with the non-diabetic (0.47 ± 0.18 nmol/l) group. 2hCP was significantly lower in the depression T2DM group (1.03 ± 0.47 nmol/l) compared with either the non-depression T2DM (1.46 ± 0.64 nmol/l) and non-diabetic (1.51 ± 0.66 nmol/l) groups. However, there were similar levels of FCP, 2hCP, and fasting ghrelin between adults without diabetes and the non-depression T2DM group. There was a significant difference in ghrelin change after meals between the non-diabetic (0.94 ± 1.09 ng/ml) and depression with T2DM (− 0.40 ± 0.97 ng/ml) groups.

As seen in Table 1, significantly larger fluctuations in blood glucose levels occurred in the depression T2DM group compared to those in the non-depression T2DM group. The depression T2DM group as compared with the non-depression T2DM group demonstrated higher MAGE, SDBG, and LAGE at 72 h. No significant between-group differences in plasma levels of fasting leptin were observed. As shown in Table 1, the depression T2DM group had the highest level of 24 h urinary cortisol and plasma cortisol levels during the three sampling times, compared to the other two groups, with the lowest fasting ghrelin levels (9.67 ± 1.93 ng/ml), the smallest decrease observed after meals in ghrelin levels (− 0.40 ± 0.97 ng/ml), the lowest 2hCP (1.03 ± 0.47 nmol/l), the highest FCP (0.61 ± 0.49 nmol/l), and the highest FBG (10.33 ± 3.49 mmol/l), being particularly higher with PHQ-9 and CES-D scores.

### Analysis of cortisol data from three sampling times in the three groups

We had an adequate sample size of 186 persons as a minimum sample size of 135 participants was required to attain 80% power for a repeated-measure ANOVA, which included one within-subjects variable (three sampling time) and one between-group variable (three groups). The post hoc power analysis showed that our tests were overpowered; all three tests achieved greater than 80% power (between factors test, the effect size was 0.20, achieving a power of 0.85 based on 186 participants at 5% alpha level; within factors test, the effect size was 0.11, achieving a power of 0.92 based on 186 participants at 5% alpha level; within-between interaction test, the effect size was 0.15, achieving a power of 0.99 based on 186 participants at 5% alpha level).

**Fig. 3:**
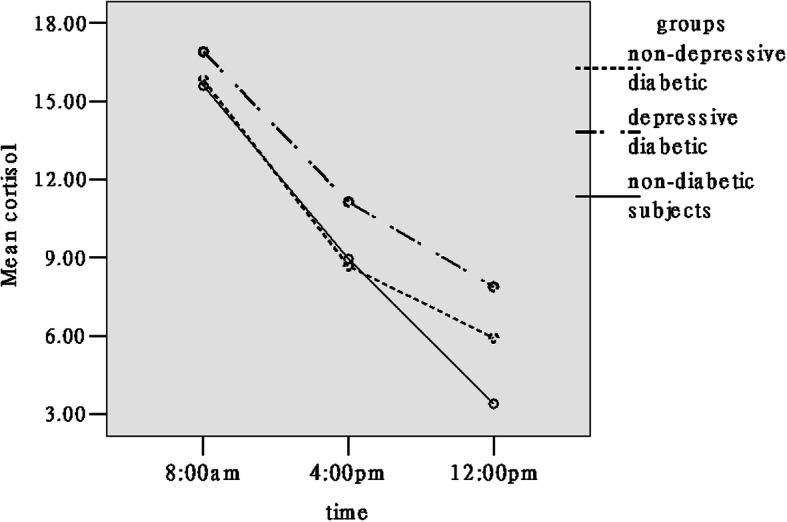
The cortisol circadian rhythm of the three groups (non-depressive, depressive diabetic, and non-diabetic)

As seen in Table 1, plasma cortisol levels at 12:00 pm were significant higher in the depression T2DM group compared with the non-depression T2DM and non-diabetic groups; and was significantly higher in the non-depression T2DM group compared with the non-diabetic group. A significant main group effect was observed in plasma cortisol concentrations (F(2,177) =14.93, *p* < 0.001). The physiological cortical rhythm curve of the depression T2DM individuals fluctuated higher than that of the non-diabetic and non-depression T2DM individuals (Fig. 3). There was similar cortisol physiological rhythm curve between the non-depression T2DM and non-diabetic groups; the only difference was in the plasma cortisol levels at 12:00 pm. An interaction between sampling time and group was found (F(4,354) = 6.24, *p* < 0.01); the cortisol levels in all individuals differed significantly over the three sampling times (all *p* < 0.01). The plasma cortisol values were not significantly different between the three groups at 8:00 am and 4:00 pm but were significantly different across all three groups at 12:00 pm.

### Multiple stepwise regression as a final model for the prediction of depressive scores

We had an adequate sample size of 136 individuals with T2DM as a minimum sample size of 69 participants was required to attain 80% power for the multiple stepwise regression (*R*^2^ increase). The post hoc power analysis showed that our tests were overpowered; both multiple stepwise regression analyses achieved a power of 1 (predictors of PHQ-9 scores, *R*^2^ increase = 0.41, the total effect size was 0.69; predictors of CES-D scores, *R*^2^ increase = 0.45, the effect size was 0.82).

The final multiple stepwise regression model (Table 2) included plasma cortisol (12:00 pm) levels, fasting ghrelin levels, and 2hCP as predictors of PHQ-9 scores. Total variance (*R*^2^ = 0.41) was explained by the following three independent variables: plasma cortisol (12:00 pm), *R*^2^ = 0.21; fasting ghrelin, *R*^2^ change = 0.11; and 2hCP, *R*^2^ change = 0.09. The final multiple stepwise regression model included 2hCP, gender, and plasma cortisol (12:00 pm) level for the prediction of CES-D scores, total variance (*R*^2^ = 0.45) was also explained by the following three independent variables: 2hCP, *R*^2^ = 0.19; gender, *R*^2^ change = 0.15; and plasma cortisol (12:00 pm), *R*^2^ change = 0.10. As shown in Table 2, a higher level of depression was associated with higher nocturnal zero plasma cortisol concentration (*p* < 0.01), lower levels of 2hCP (*p* < 0.01), lower fasting ghrelin level (*p* < 0.01), and female sex (*p* < 0.01). Age, BMI, education level, duration of diabetes, history of hypertension, FBG, HbA1c, fasting leptin, plasma cortisol at 8:00 am, plasma cortisol at 4:00 pm, 24 h urinary cortisol, and FCP were not significantly correlated with depression.

**Table 2:** Multiple stepwise regression models for all patients with T2DM

Dependent variable: PHQ-9 total scores					Dependent variable: CES-D total scores			
Independent variables	B	Beta	t	R Square	B	Beta	t	R Square
	(Std. Error)			Change	(Std. Error)			Change
Plasma cortisol (12:00 pm)	0.19(0.07)	0.38	2.71**	0.21	0.62(0.20)	0.43	3.19**	0.10
Fasting ghrelin	−1.54(0.51)	−0.31	−2.99**	0.11				
2hCP	−2.55(0.93)	−0.30	−2.74**	0.09	−4.79(1.43)	−0.50	−3.35**	0.19
Gender					4.78(1.63)	0.45	2.93**	0.15

*T2DM* Type 2 diabetes mellitus, *PHQ-9* Patient Health Questionnaire-9, *CES-D* Center for Epidemiological Studies Depression scale, *Std Error* Standard error, *2hCP* 2 h postprandial C-peptide

**There was a significant correlation (*p* < 0.01). Gender (1-male, 2-female)

## Discussion

A post hoc power analysis showed that we had an adequate sample size and met the minimum requirement to attain 80% power. A post hoc power calculation also demonstrated that this study basically achieved power of 80% at 5% alpha level.

This study assessed depression symptoms and biochemical indicators(levels of ghrelin, leptin, cortisol, and C-peptide levels) in type 2 diabetes mellitus (T2DM). The level of 2 h of C-peptide (2hCP) was negatively correlated with depression level, and elevated evening cortisol was associated with higher levels of depression. Our results also confirmed prior research that shows a positive correlation between cortisol levels and depression [34]. Leptin levels were not associated with depression. Altogether, these findings lead to a more complete picture of biomarkers associated with depression in T2DM. We believe that our study makes a significant contribution to the literature because no previous studies have explored the association between C-peptide levels and depression in diabetics.

Fasting levels of ghrelin in the depression T2DM group were significantly lower than that for the non-depression T2DM and non-diabetic groups (Table 1), while lower fasting ghrelin level were associated with higher levels of depression (Table 2). This illustrates a possible long-term effect of ghrelin on HPA axis function and ultimately depressive symptomology [21, 45]. This supports previous studies showing that ghrelin levels are decreased in patients with depression [24]. However, other studies are not consistent. Compared to healthy individuals, ghrelin levels in patients with major depressive disorder (MDD) were found to be higher in one study [25], and comparable in the other [26]. Compared with the non-diabetic group (− 0.94 ± 1.09 ng/ml), the decrease of ghrelin level at 2 h postprandial in depression T2DM group (− 0.40 ± 0.97 ng/ml) became significantly lower. Similarly, Paslakis et al. found that ghrelin levels displayed a significantly blunted response to a standard glucose load in patients with MDD when compared to healthy controls [46]. Many previous studies have regarded ghrelin as a marker of physical and mental balance [18, 47]. In this sense, depressed diabetics exhibit greater mental and physical imbalance. Therefore, the lower fasting ghrelin levels seen in depression T2DM patients may be a double manifestation of poor islet function and depression. A previous study, which discussed animal and human studies, showed that ghrelin regulates the HPA axis and affects anxiety and mood disorders, such as depression and fear-related behaviors. They concluded that ghrelin has a potential role in providing a stress feedback signal that regulates these associated behaviors [18].

Previous studies have shown that FBG [48] and HbA1c were significantly higher in depression T2DM patients than in non-depression T2DM patients [49]; however, our study showed no significant difference in FBG and HbA1c between the two groups. As shown in Table 1, depression T2DM patients had lower 2hCP than non-depression T2DM patients. Use of a glucose monitoring system for 72 h afforded continuous dynamic monitoring of the patients and revealed higher MAGE, SDBG, and LAGE, which showed larger fluctuations in blood glucose levels, correlating with higher levels of depression. The unique finding in our study was that depression T2DM patients had lower postprandial C-peptide, but there was no significant difference in non-depression T2DM patients and non-diabetic individuals. A recent study demonstrated that random non-fasting C-peptide levels can be used to indicate hypoglycemia risk in insulin-treated T2DM individuals. Their results demonstrate that patients with insulin-treated T2DM with low postprandial C-peptide levels have markedly increased incidence of hypoglycemia in comparison to those with retained C-peptide levels [13]. The observation from that study is in full agreement with the conclusion from our study. These findings indicate that the postprandial C-peptide levels were low, which makes hypoglycemia risk and promotes blood sugar fluctuations. According to the relevant endocrine theory, the increase in FCP and decrease in 2hCP reflect impaired islets function. Since the C-peptide was not affected by exogenous insulin, the C-peptide can thus reflect islet function to some extent [12]. Previous studies have shown that islet function is closely related to glycemic control in patients with T2DM, indicating that lower beta cell function is associated with greater postprandial glycemic excursions in patients with T2DM [50].

It may be that the larger fluctuations in blood glucose levels resulted in increased panic and hence increased levels of depression in patients with diabetes. Clinically, the treatment of diabetes depends on the islet function of the patient. If the islet function is adequate, the patient produces less endogenous insulin when using more hypoglycemic drugs, injecting exogenous insulin, or eating less food. Conversely, more insulin is produced when a patient takes less hypoglycemic drugs, injects less insulin, or eats more food, resulting in more stable blood sugar levels. With significant islet function loss, there is a corresponding impairment in the ability to regulate, in combination with external treatment, and thus blood sugar fluctuations become greater, increasing the likelihood of panic in patients with diabetes. The change of C-peptide levels in depression T2DM patients is consistent with the larger fluctuations in blood glucose.

In our study, multiple stepwise regression analysis did not reveal a significant association between leptin and depression. Further, there was no significant difference in leptin levels between the depressive and non-depressive individuals with T2DM. This could be because leptin is secreted by adipocytes and is associated with BMI, which showed no significant between-group difference. There are inconsistent previous findings regarding the relationship between depression and leptin with studies reporting higher, lower, and similar leptin levels in individuals with depression compared with those without depression [51–53]. Table 2 shows that a higher level of depression was associated with the female gender, this conclusion has been confirmed by many studies [4, 54, 55].

We found a significant increase in cortisol secretion in the depressive T2DM group, and higher 12:00 pm cortisol levels, which was associated with higher levels of depression. The circadian rhythm curve of depression T2DM patients floated higher on top, compared with the non-depressive group with diabetes and the non-diabetic group. Cortisol levels in the depression T2DM patients, at all three time-points, were the highest. The cortisol daily rhythm indicates the resting-state activity pattern of the HPA axis in depression T2DM patients differed more from that in the non-diabetic group [33]. From Fig. 2, we can speculate that diabetes and depression in the depression T2DM patients might have resulted from a considerable number of daily life challenges, causing a long-term high stress emotional state. Cortisol levels of all individuals differed significantly over the three sampling times (all *p* < 0.01). Previous studies found that cortisol levels at 4:00 pm and 12:00 pm fell flat on the steep slope and did not differ significantly over the two sampling times relating to the physiological rhythm of cortisol in elderly individuals with depression [56]. Our study participants were middle-aged as compared with elderly depressed patients, and the course of depression was shorter. As a result, middle-aged depressive T2DM patients had less impaired cortisol rhythm than elderly depressive individuals. Figure 2 showed significantly higher 12:00 pm cortisol levels in the depressive patients with diabetes compared with the non-diabetic group and the non-depressive group with diabetes. Previous studies have shown that high levels of cortisol at 12:00 pm were associated with higher levels of depression [57]. In addition, it can be seen that 12:00 pm cortisol levels in non-depressive individuals with diabetes is also significantly higher than that in non-diabetic patients, which indicates that diabetes does increase psychological stress. Cortisol levels at the other two time-points (the morning and afternoon) were similar to those in the non-diabetic group. From previous analyses, we can infer that more functional losses cause long-term psychological stress in depressive patients with diabetes. Kathol et al. has reported that exposure to chronic stress increases the activity of the HPA axis and depression level [58]. Previous studies have shown a two-way relationship between depression and diabetes, such that diabetes increases the level of depression, which in turn reduces the self-caring compliance of patients with diabetes. This negatively affects diet, exercise and medication or insulin therapy compliance further exacerbating the diabetes [59].

There are several limitations to this study that must be considered. The main limitation is that since we used a constructed interview, the sample size was relatively small and therefore limited. However the results of a post hoc power analysis showed that we had an adequate sample size and met the minimum requirement to attain 80% power. A post hoc power calculation also demonstrated that this study basically achieved power of 80% at 5% alpha level. It would have been useful to add a group of patients with depression and not diabetes as this may have provide a more comprehensive view on how diabetes and depression affect hormone levels. Additionally, sampling C-peptide at multiple time points (samples taken 1–5 h post meal) would provide more detail and allow for a more scientific and reliable assessment of all the variables.

## Conclusions

Overall, the depressive patients with T2DM showed decreased levels of 2hCP, and increased levels of cortisol compared to the non-depressive group with T2DM. Leptin levels, however, were unremarkable in all groups. Lower levels of 2hcp and higher levels of plasma cortisol (12:00 pm) were correlated with higher levels of depression. Thus, these findings suggest that 2 h C-peptide, and evening cortisol levels may be markers of depression in patients with T2DM. Recognizing these will improve management of diabetes.

## Data Availability

The study data is available from the corresponding author upon request.

## Abbreviations

2hCP: 2 h postprandial C-peptide
BMI: Body mass index
CES-D: Center for Epidemiologic Studies Depression Scale
FBG: Plasma glucose before the meal
FCP: Fasting C-peptide
HbA1c: Glycated hemoglobin
HPA axis: Hypothalamic-pituitary-axis
LAGE: Large amplitude of glycemic excursions
MAGE: Mean amplitude of glucose excursions
MBG: The day time mean values blood glucose
PBG: Postprandial blood glucose
PHQ-9: Patient Health Questionnaire-9
SDBG: Standard deviation blood glucose
